# Preparation and Characterization of Antioxidative and UV-Protective Larch Bark Tannin/PVA Composite Membranes

**DOI:** 10.3390/molecules23082073

**Published:** 2018-08-19

**Authors:** Yingxiang Zhai, Jiangtao Wang, Hao Wang, Tao Song, Weitong Hu, Shujun Li

**Affiliations:** College of Materials Science and Engineering, Northeast Forestry University, Harbin 150040, China; 18846831107@163.com (Y.Z.); 18845893570@163.com (J.W.); 15844451795@163.com (H.W.); 15603662305@163.com (T.S.); h17745136302@163.com (W.H.)

**Keywords:** larch bark tannin, composite membrane, antioxidation, UV protection

## Abstract

In order to prepare functional materials for antioxidant and ultraviolet (UV)-protective green food packaging, condensed tannin, previously extracted from larch bark, was mixed with polyvinyl alcohol (PVA), and then the mixture was used to cast composite membranes. An antioxidative assay using 1,1-diphenyl-2-picrylhydrazyl (DPPH)—a free radical scavenger—and starch–potassium iodide oxidation–discoloration analyses showed that the composite membranes have good antioxidative activities. The low UV transmission and protective effect of the composite films on vitamin E indicated the UV protection ability of the composite membranes. Both larch bark tannin and PVA are rich in hydroxyl groups; scanning electron microscopy analysis demonstrated their compatibility. Also, the mechanical and crystallization properties of the composite membranes did not significantly decrease with the addition of larch bark tannin.

## 1. Introduction

Food packaging is an important component of food products. Food easily oxidizes and deteriorates. Antioxidation is a key function of active packaging materials, which, therefore, are helpful for extending the shelf life of foods. Ultraviolet (UV) light can also promote the oxidative degradation of foods. Developing food packaging materials with antioxidative and anti-UV radiation properties is essential. Considering the direct contact of certain packaging materials with food, natural products may be preferable for food packaging materials. Examples of natural antioxidants currently used for food packaging include extracts of apple pomace, guarana, rosemary, and cinnamon bark [1,2].

Vegetable polyphenols are well known as natural antioxidants and they also have anti-UV properties. Polyphenols are important secondary metabolites and are widely present in plants [3]. Larch (*Larix gmelinii*), which is a conifer abundant in temperate zones of the northern hemisphere, contains a large amount of polyphenols in its bark [4]. The polyphenols in larch bark, which include condensed tannin, are highly polymerized compared with other condensed vegetable polyphenols. Although this raw material is inexpensive and easily obtained, larch bark tannin (LBT) has not been extensively used [5,6].

Polyvinyl alcohol (PVA) is a highly polar and high-strength biodegradable polymer with good thermal stability and membrane-forming ability, which makes it ideally suited for blending with natural polyphenols [7,8]. Accordingly, in this work, LBT was extracted and then mixed with PVA to prepare composite membranes. The antioxidative and anti-UV radiation properties of the membranes were then evaluated. The knowledge derived from this study provides a good foundation for the high-added-value application of LBT.

## 2. Results and Discussion

### 2.1. Analysis of Membrane Antioxidative Activity

**Figure 1:**
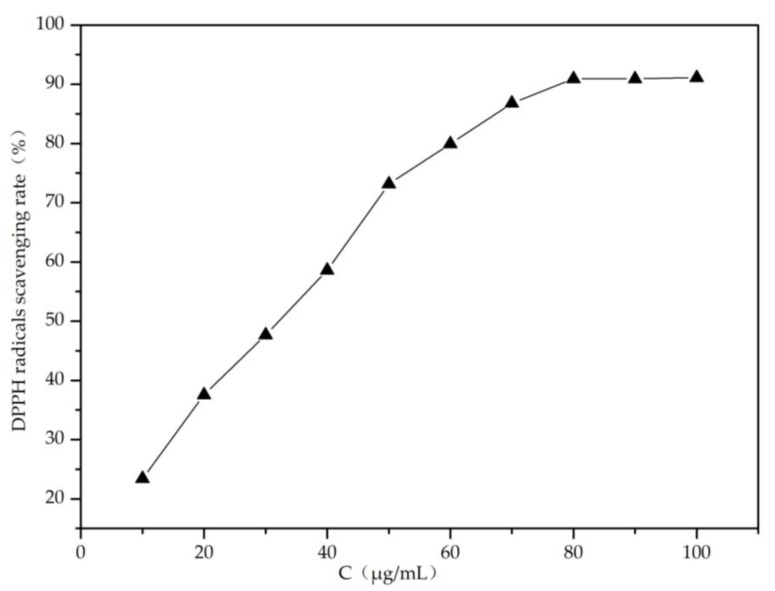
Rate of 1,1-diphenyl-2-picrylhydrazyl (DPPH) radical scavenging by larch bark tannin (LBT) in solution.

**Figure 2:**
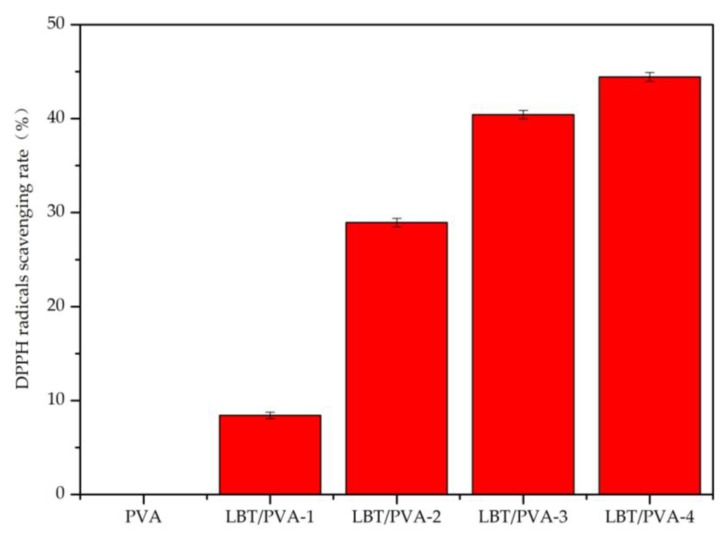
Scavenging rate of DPPH radicals in LBT/Polyvinyl alcohol (PVA) membranes. PVA, LBT/PVA-1, LBT/PVA-2, LBT/PVA-3, and LBT/PVA-4 represent composite membranes with LBT content of 0.00, 0.25, 0.50, 1.00, and 2.00%, respectively.

Because of its polyphenols content, LBT presented high 1,1-diphenyl-2-picrylhydrazyl (DPPH) radical scavenging ability in solution (Figure 1). In the composite LBT/PVA membrane, LBT endowed the membrane with good DPPH radical scavenging ability. When the membrane came into contact with moisture, water molecules entered it, causing the swelling of the film. As a result, some LBT molecules were released from the composite membranes. Over time, the network structure of the membrane loosened and the maintenance energy decreased, resulting in a dramatic increase in the release of LBT, which enhanced DPPH free radical scavenging [9]. As shown in Figure 2, the scavenging rate of DPPH free radicals increased significantly with the increase of the tannin content in the LBT/PVA composite membrane. When the tannin content increased from 0.25 to 2.00%, the scavenging rate of DPPH radicals increased from 8.43 to 44.44%.

### 2.2. Membrane Soluble Phenols Content

**Figure 3:**
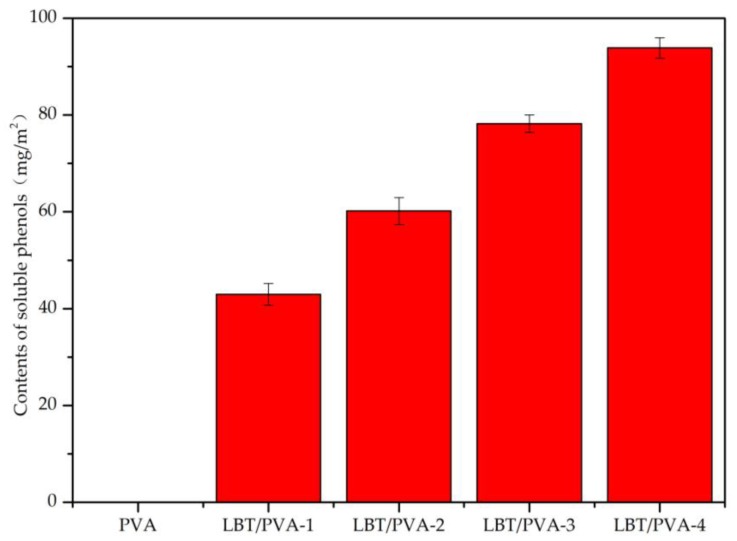
Contents of soluble phenols in the composite LBT/PVA membranes.

In order to evaluate the effects of different concentrations of LBT in the membrane, soluble phenols in the membranes were determined, and the result is illustrated in Figure 3. With the increase of LBT concentration in the membranes, the content of soluble phenols increased. This resulted in improved DPPH radical scavenging, antioxidation in the acidic starch–potassium iodide (KI) discoloration reaction, and UV protection. However, the increase in soluble phenol content was not proportional to the LBT content. The reason for this was that membranes with different LBT contents were incubated for the same limited time (30 min), and the release profiles of soluble phenols were similar in this short time frame. Additionally, the release rate was influenced by other factors besides the initial LBT content, such as diffusion area and diffusion distance.

### 2.3. Effect of Increasing Amounts of LBT in the Membrane on Starch-KI Oxidation–Discoloration Reaction

**Figure 4:**
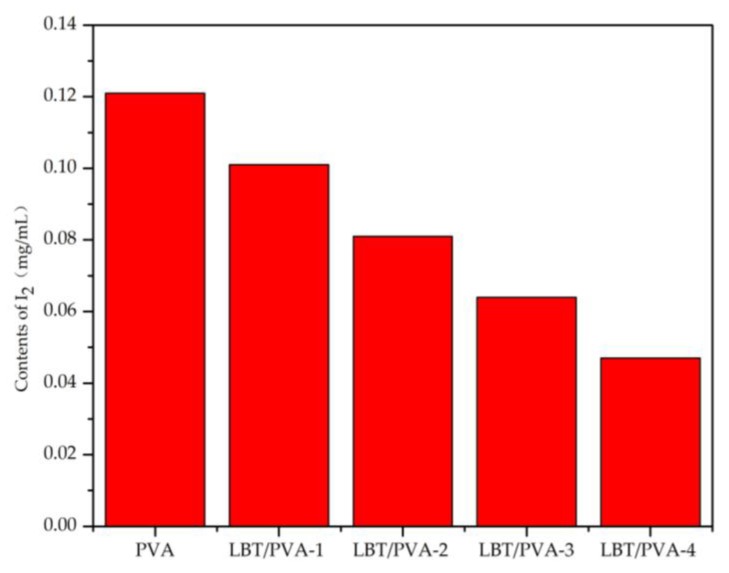
The effect of tannin content in the composite membrane on the amount of I_2._

With the increase of LBT content in the composite membranes, the membranes became increasingly resistant to oxidation. The presence of LBT inhibited I^−^ oxidation to I_2_, thereby reducing the amount of I_2_ generated. According to Figure 4, the amount of I_2_ produced with the blank PVA sample was the highest, corresponding to about 0.12 mg/mL. With increasing amounts of LBT in the composite membrane, the content of I_2_ gradually decreased, reaching a minimum (about 0.05 mg/mL) in the LBT/PVA-4 membrane. This implies that the higher the LBT content in the composite membrane, the stronger the antioxidant capacity of the membrane.

### 2.4. Light Transmittance of the Composite Membranes

**Figure 5:**
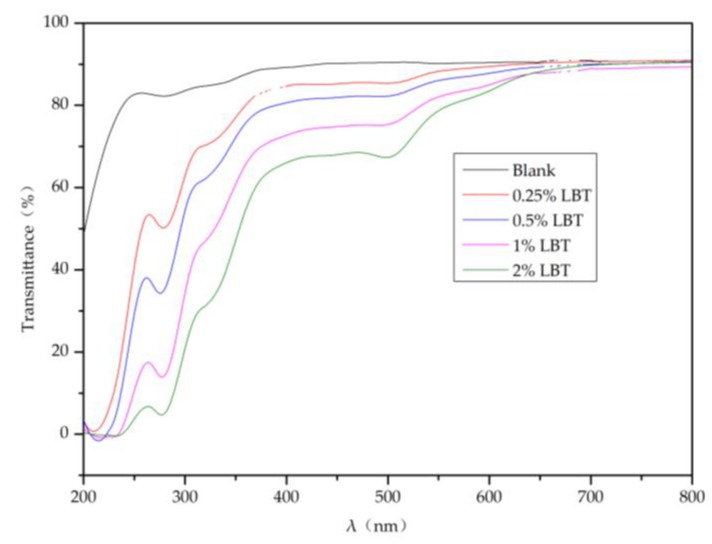
Ultraviolet–visible (UV–vis) light transmittance of the composite membranes.

The data presented in Figure 5 indicate that with increasing tannin content in the composite membrane, the transmittance of UV light considerably decreased, meaning that UV absorption by the membrane increased accordingly [10]. As the tannin content increased from 0 to 2.00%, the transmittance of UV light (wavelength less than 280 nm) decreased from 80% to less than 10%, while the transmittance of near-infrared (650 to 800 nm) light did not decrease. The transmittance of visible (400–500 nm) light also decreased from 90 to ~65%. This indicates that the composite membrane is suitable for UV-protective packaging materials.

### 2.5. Analysis of the Mechanical Properties of Membranes

**Figure 6:**
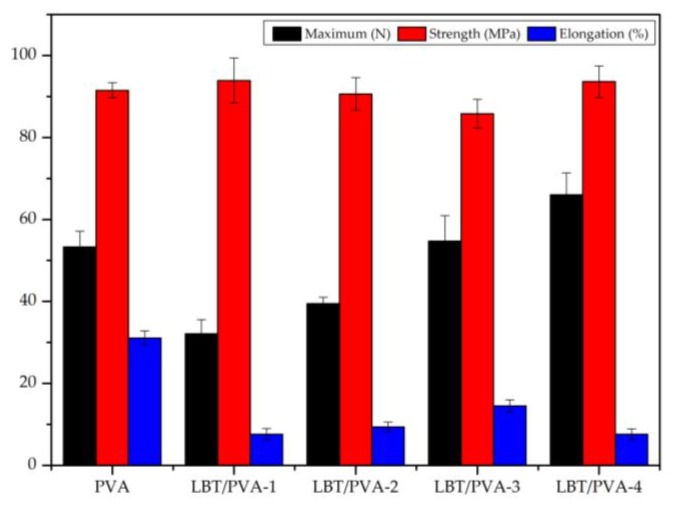
Mechanical properties of the composite membranes.

The results shown in Figure 6 reveal that the average maximum force of the blank membrane was 53.32 N. After the addition of 0.25% LBT, the average maximum force of the membrane decreased to 32.12 N. PVA molecules are linear polymers, which could easily organize into aggregates. When a small amount of LBT was added to the PVA matrix, the well-organized aggregate structure of PVA was altered, resulting in a decrease in its strength. However, with the increase of LBT content, the maximum force of the membrane increased because of the good compatibility of LBT with PVA. For example, with the addition of 2.00% LBT, the average maximum force reached 66.03 N. Compared to the maximum force, the tensile strength of the membranes did not change much with the increase of LBT content. The tensile strength was about 90 MPa for all membrane samples. Regarding elongation, the value for the blank membrane was 31.1% and decreased considerably with the addition of 0.25–2.00% LBT. The reason for this could be that the tannin molecules cross-linked, thus limiting the rotation and movement of the PVA molecular chains, which resulted in the elongation decrease [11].

### 2.6. Other Analytical Characterization

**Figure 7:**
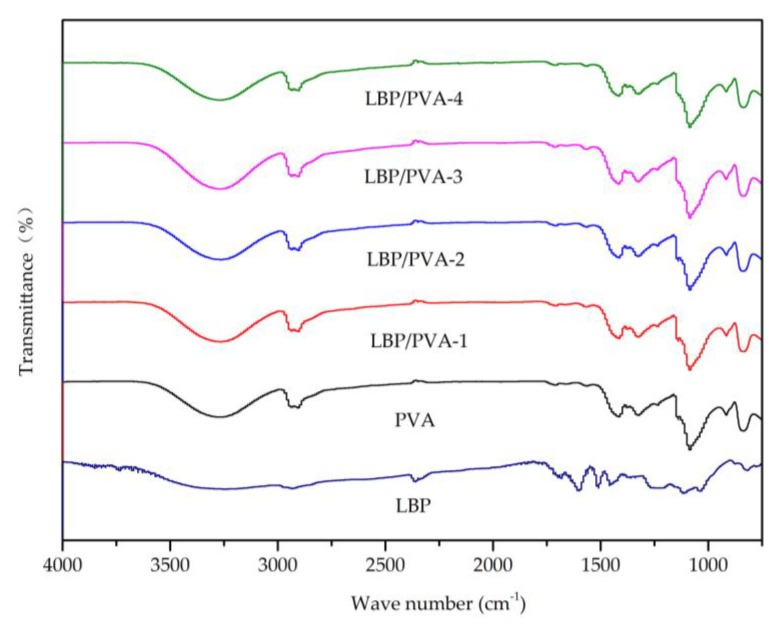
Fourier transform–infrared (FT-IR) spectra of LBT, PVA, and LBT/PVA membranes.

The FT–IR spectra of the PVA, LBT, and LBT/PVA composite membranes with different LBT contents are displayed in Figure 7. The broad peak at about 3300 cm^−1^ was attributed to the hydroxyl group (–OH). The small peak at about 2900 cm^−1^ was attributed to the stretching vibration of C–H. The peak at about 1100cm^−1^ was ascribed to the stretching vibration of the ether bond (C–O). Because of the very low amount of LBT in the composite membranes (the maximum amount was only 2.00%), the FT–IR spectra of the composite membranes did not show any obvious peaks corresponding to LBT [12].

**Figure 8:**
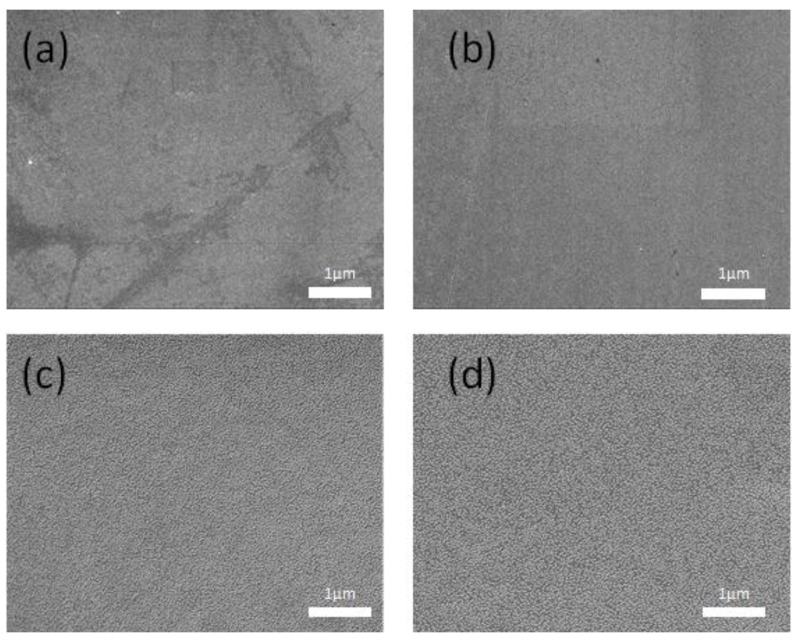
Scanning electron microscopy (SEM) images of the surface of (**a**) PVA and (**b**) LBT/PVA-2 films and cross section of (**c**) PVA and (**d**) LBT/PVA-2 films.

The SEM images (Figure 8) showed that tannin was evenly distributed in the PVA matrix. Both tannin and PVA molecules are rich in hydroxyl groups, which results in their high polarity and good compatibility [13].

**Figure 9:**
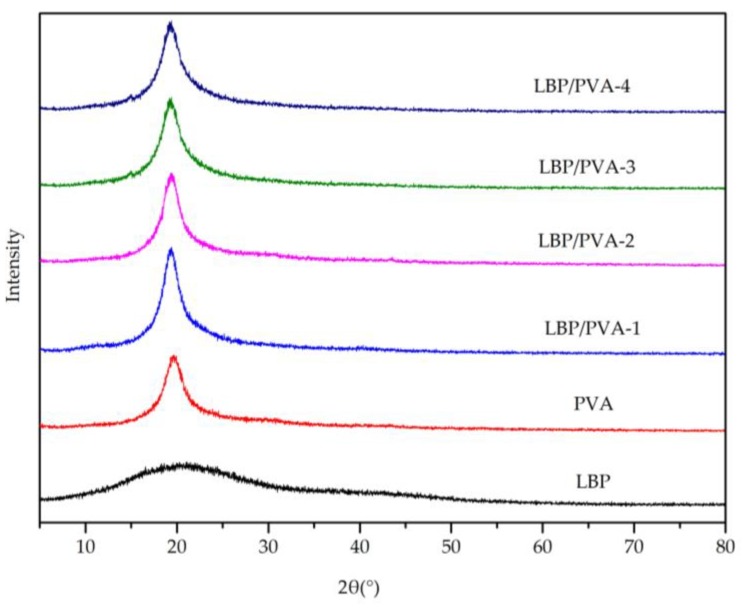
X-ray diffraction (XRD) patterns of the LBT, PVA, and LBT/PVA composite membranes.

The PVA molecular chains contain a large number of -OH groups, which easily form hydrogen bonds both intra- and inter-molecularly. Moreover, the ordered molecular long chains arranged by hydrogen bonds can form a dense structure. The XRD patterns of LBT, PVA, and LBT/PVA composite membranes (Figure 9) showed one sharp peak at a 2θ angle of 19.6° for the pure PVA membrane, due to the membrane’s partially ordered and dense structure. Compared with PVA, LBT is amorphous. With addition of LBT (0.25 to 2.00%), the partially ordered and dense structure of PVA was not changed much, as shown by the values of both the 2θ angle and the intensity, which meant that the addition of LBT did not obviously damage the orderly arranged structure [14].

### 2.7. Application for Vitamin E Protection

**Figure 10:**
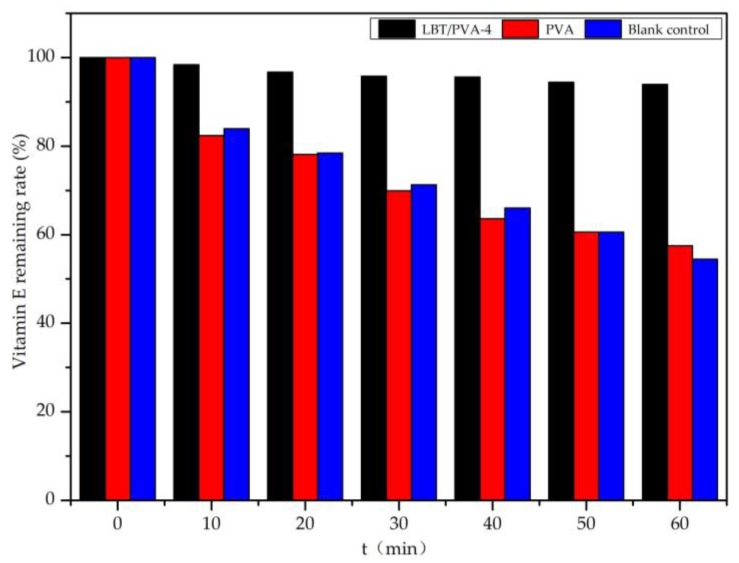
Protective effect of the LBT/PVA-4 membrane on Vitamin E.

According to Figure 10, without coating protection, vitamin E was degraded by UV irradiation very quickly, with nearly 20% vitamin E loss in the first 10 min. After 60 min of irradiation, the remaining vitamin E was only 60% of the original amount. When the PVA membrane without LBT was used for coating protection, the rate of vitamin E degradation was similar to that of the blank control. However, when using LBT/PVA-4 for coating protection, vitamin E was not degraded significantly. Even after 60 min of irradiation, only 5.0% of vitamin E was lost. We concluded that the composite membrane was helpful in protecting vitamin E from degradation by UV light.

## 3. Materials and Methods

### 3.1. Materials

Larch bark from *Larix gmelinii* (Rupr.) Kuzen was ground and sifted through 40-mesh sieves. The PVA had an average degree of polymerization of 1750 ± 50 and a sodium acetate content of less than 0.5%. Gallic acid and other reagents were all analytical-grade.

### 3.2. Extraction of Tannin from Larch Bark

LBT was extracted using an ethanol/water (1:1, *v/v*) mixture as the solvent, with a solid/liquid ratio of 1:20, at a temperature of 55 °C, for 2.5 h. After filtration, the filtrate was evaporated and vacuum-dried for use. The yield was about 10.52 g tannin/100 g larch bark. Using the gallic acid standard curve method, the total polyphenol content of LBT was determined as 545.76 mg/g [15].

### 3.3. Preparation of the Composite Membrane

PVA was first dissolved in water (5% *w/w*) at 90 °C [16]. Then, an LBT ethanol/water (50% *v/v*) solution was added into the PVA water solution at room temperature with agitation, and the homogeneous mixture was casted into membranes. According to their LBT content of 0.00, 0.25, 0.50, 1.00, and 2.00%, the membranes were designated as PVA, LBT/PVA-1, LBT/PVA-2, LBT/PVA-3, and LBT/PVA-4, respectively.

### 3.4. Determination of the Antioxidative Activity of LBT and Composite Membranes by the DPPH Assay

An alcohol solution of DPPH free radicals is purple and has a strong absorption at 517 nm. In the presence of some free radical scavengers, DPPH free radicals will be scavenged, and the absorption gradually decreases. There is a quantitative relationship between absorption and DPPH radical scavenging, so this assay is considered one of the standard methods for the evaluation of antioxidative substances [17].

In this work, LBT was dissolved in 50% (*v/v*) ethanol/water and used for this test. The membrane samples were also used for this test according to the following method.

The membrane samples were cut into 1 × 1 cm pieces, immersed in a sample bottle containing 10 mL distilled water, and stirred at room temperature for 30 min. Then, 1 mL of the solution was added to 4 mL of DPPH solution (150 μmol/L), and the solution was mixed by agitation. The mixture was placed in the dark for 30 min to allow the DPPH free radicals to be fully scavenged. Then, a UV spectrophotometer was used to determine the absorbance of the solution at 517 nm [18]. The scavenging rate of DPPH free radicals was calculated according to Equation (1):

DPPH radicals scavenging rate (%) = (1 − A_sample_/A_blank_) × 100 (1)

where A_sample_ is the absorbance of the sample and A_blank_ is the absorbance of the blank.

### 3.5. Determination of Soluble Phenol Content

The soluble phenol contents in the composite membranes were determined according to the following method. The first step of the membrane DPPH assay was adopted to produce a phenol solution from LBT/PVA composite membrane samples. The membrane samples were cut into 1 × 1 cm pieces, placed in a sample bottle containing 10 mL distilled water, and stirred in a magnetic stirrer at room temperature for 30 min; then, 1 mL of the solution was added into a 10 mL volumetric flask with 10% 5 mL formol reagent. After 5 min of reaction, 4 mL of 75% (*w/w*) sodium carbonate solution was added. The absorbance value was measured at 765 nm after placing the samples in a water bath for 30 min. The contents of soluble phenols in the composite membranes were calculated on the basis of a standard curve prepared with gallic acid [19].

### 3.6. Effect of the Composite Membranse on the Oxidation and Discoloration of Starch-Potassium Iodide (KI)

Iodide ions (I^−^) can be oxidized into I_2_ by the oxygen in the air under acidic conditions, through the reaction shown in Equation (2), and the I_2_ produced will react with aqueous starch and turn it blue. In the presence of the composite membrane, the amount of I_2_ produced varied, depending on the antioxidative properties of the membranes.

4 I^−^ + O_2_ + 4 H^+^ → 2 I_2_ + 2 H_2_O (2)

The membrane samples were cut into 2 × 2 cm pieces, separately immersed in a sample bottle with 10 mL distilled water, and stirred for 30 min at room temperature. Then, 2 mL of each membrane solution was mixed with 2 mL of KI (1 mg/mL), and then 20 μL of 3% H_2_O_2_ was added to a pH value of 5.5. After 2 h, each solution was titrated with sodium thiosulfate to determine the content of I_2_ [20].

2 S_2_O_3_^2−^ + I_2_ → S_4_O_6_^2−^ + 2 I^−^ (3)

### 3.7. UV–Visible Light Transmittance of the Composite Membranes

A TU-1901 double beam UV–visible spectrophotometer (Persee Universal Instrument Co., Beijing, China) was used to determine the transmittance (*T* %) of the composite membranes (50 ± 5 μm in thickness) in a scanning range of 200 to 800 nm.

### 3.8. Mechanical Properties Test

The membrane samples were cut into 50 × 10 mm pieces. The maximum force (N), tensile strength (MPa), and elongation rate at break (%) of the membranes under dry conditions were tested with a small mechanical test machine (Landmark Sci. & Tech. Co., Beijing, China) at a speed of 20 mm/min. All tests were performed in quintuplicate, and the results expressed as mean ± standard deviation [21].

### 3.9. Other Analysis and Characterizations

Fourier transform–infrared (FTIR) spectra were recorded with a Nicolet iS10 FT-IR spectrometer (Thermo Fisher Scientific, Waltham, MA, USA) using the attenuated total reflection (ATR) method [22]. The scanning range was from 500 to 4000 cm^−1^, and the resolution was 4 cm^−1^.

Scanning electron microscopy (SEM) micrographs of the samples were taken with a Quanta 200 scanning electron microscope (FEI Co., Hillsboro, OR, USA), with an accelerating voltage of 10 kV. The membranes were frozen in liquid nitrogen and snapped immediately to prepare the cross sections. Prior to observation, the samples were coated with a thin gold layer [23].

X-ray diffraction analysis of LBT and the composite membranes was performed on a D/Max-2400 diffractometer (Rigaku Corporation, Tokyo, Japan) using a scanning angle of 5–80° at a speed of 5°/min [24].

### 3.10. Protective Effect of the Composite Membranes on Vitamin E

Vitamin E is a common nutrient in food that oxidizes easily and deteriorates under the irradiation of ultraviolet light [25]. Using an LBT/PVA composite membrane as a food packaging material could protect vitamin E, since the LBT/PVA composite membranes demonstrated excellent UV protection ability. In this paper, a quartz cuvette containing 40 ppm vitamin E *n*-hexane solution was coated with one of the membranes, and then the solution was irradiated with a 3 W ultraviolet lamp at 254 nm. A non-coated sample served as the blank control. The absorbance of the vitamin E solution was measured at 300 nm, with 10 min irradiation intervals, and the remaining vitamin E was calculated on the basis of the prepared standard curve.

## 4. Conclusions

The condensed tannin fraction that was extracted from larch bark was successfully used to prepare composite membranes with PVA and provided the PVA membranes with antioxidative and anti-UV properties. The DPPH free radical scavenging and starch-KI oxidation–discoloration assays showed that the composite membranes had good antioxidative ability. The low UV transmittance and the protective effect of the composite membrane on Vitamin E indicated that the composite membrane with 2.00% LBT provided excellent UV protection. The addition of the LBT did not cause obvious changes in the mechanical strength and crystallization properties of the membranes due to the good compatibility of tannin with PVA. The LBT/PVA composite membranes have the potential to be used as antioxidative and UV-protective food packaging.
